# A Comprehensive Literature Review on the Clinical Presentation, and Management of the Pandemic Coronavirus Disease 2019 (COVID-19)

**DOI:** 10.7759/cureus.7560

**Published:** 2020-04-06

**Authors:** Pramath Kakodkar, Nagham Kaka, MN Baig

**Affiliations:** 1 Medicine, National University of Ireland Galway, Galway, IRL; 2 Orthopaedics, University Hospital Galway, Galway, IRL

**Keywords:** covid-19, sars-cov-2, severe acute respiratory infection, pandemic, mrna-1273 vaccine, remdesivir (gs-5734), chloroquine, ards, ace2, lopinavir and ritonavir

## Abstract

Coronavirus disease 2019 (COVID-19) is a declared global pandemic. There are multiple parameters of the clinical course and management of the COVID-19 that need optimization. A hindrance to this development is the vast amount of misinformation present due to scarcely sourced manuscript preprints and social media. This literature review aims to presents accredited and the most current studies pertaining to the basic sciences of SARS-CoV-2, clinical presentation and disease course of COVID-19, public health interventions, and current epidemiological developments.

The review on basic sciences aims to clarify the jargon in virology, describe the virion structure of SARS-CoV-2 and present pertinent details relevant to clinical practice. Another component discussed is the brief history on the series of experiments used to explore the origins and evolution of the phylogeny of the viral genome of SARS-CoV-2. Additionally, the clinical and epidemiological differences between COVID-19 and other infections causing outbreaks (SARS, MERS, H1N1) are elucidated.

Emphasis is placed on evidence-based medicine to evaluate the frequency of presentation of various symptoms to create a stratification system of the most important epidemiological risk factors for COVID-19. These can be used to triage and expedite risk assessment. Furthermore, the limitations and statistical strength of the diagnostic tools currently in clinical practice are evaluated. Criteria on rapid screening, discharge from hospital and discontinuation of self-quarantine are clarified. Epidemiological factors influencing the rapid rate of spread of the SARS-CoV-2 virus are described. Accurate information pertinent to improving prevention strategies is also discussed.

The penultimate portion of the review aims to explain the involvement of micronutrients such as vitamin C and vitamin D in COVID19 treatment and prophylaxis. Furthermore, the biochemistry of the major candidates for novel therapies is briefly reviewed and a summary of their current status in the clinical trials is presented. Lastly, the current scientific data and status of governing bodies such as the Center of Disease Control (CDC) and the WHO on the usage of controversial therapies such as angiotensin-converting enzyme (ACE) inhibitors, nonsteroidal anti-inflammatory drugs (NSAIDs) (Ibuprofen), and corticosteroids usage in COVID-19 are discussed.

The composite collection of accredited studies on each of these subtopics of COVID-19 within this review will enable clarification and focus on the current status and direction in the planning of the management of this global pandemic.

## Introduction and background

History of the outbreak

On 31st December 2019, Wuhan health commission in the Hubei province of the Republic of China notified the National Health Commission, China CDC and WHO of a cluster of 27 cases of pneumonia of unknown etiology [1]. These patients presented with a constellation of symptoms such as fever, dyspnea, dry cough, and radiological findings showed bilateral lung glassy opacities. Furthermore, the public health office traced all these 27 cases to Huanan Seafood Wholesale Market which trades live species of bats, snakes, pangolins, and badgers [1]. Multiple intrinsic variables led to rapid early transmission dynamics, and this made Wuhan the flashpoint of the pandemic. In 2018, Wuhan had a documented population of 11.08 million, this made Wuhan one of the top five most populated cities in China [2]. Wuhan’s large population density and proximity of the marketplace that sold live animals made it the epicenter for the human-animal interface. Additionally, the lack of early containment due to the inability to accurately trace the history of exposure in the early patient cases contributed to the rapid rate of spread in Wuhan. This eventually precipitated into the WHO declaring this viral pneumonia as an outbreak on 30th January 2020. On 11th March 2020, due to the global logarithmic expansion of the cases the coronavirus disease 2019 (COVID-19) was declared as a pandemic by the WHO.

Virology

On the 7th January 2020, the China CDC discovered the virus called novel coronavirus 2019 (2019-nCoV) which was colloquially noted as the “Wuhan Coronavirus”. The WHO renamed it to SARS-CoV-2 to destigmatize the association of the virus with any geographic location or nationality and relate it to the disease symptomatology. The SARS-CoV-2 virus is genetically similar to the SARS Coronavirus of 2002 (SARS-CoV-1). There are a myriad of other coronaviruses that cause the common cold. These coronaviruses can become infective when they attain an animal reservoir that provides an adequate cellular environment wherein the virus can multiply and acquire a series of advantageous genetic mutations. These mutations can then enable the virus to cross-species and infect and multiply within human hosts effectively.

The virion structure and pathophysiology of infection

**Figure 1:**
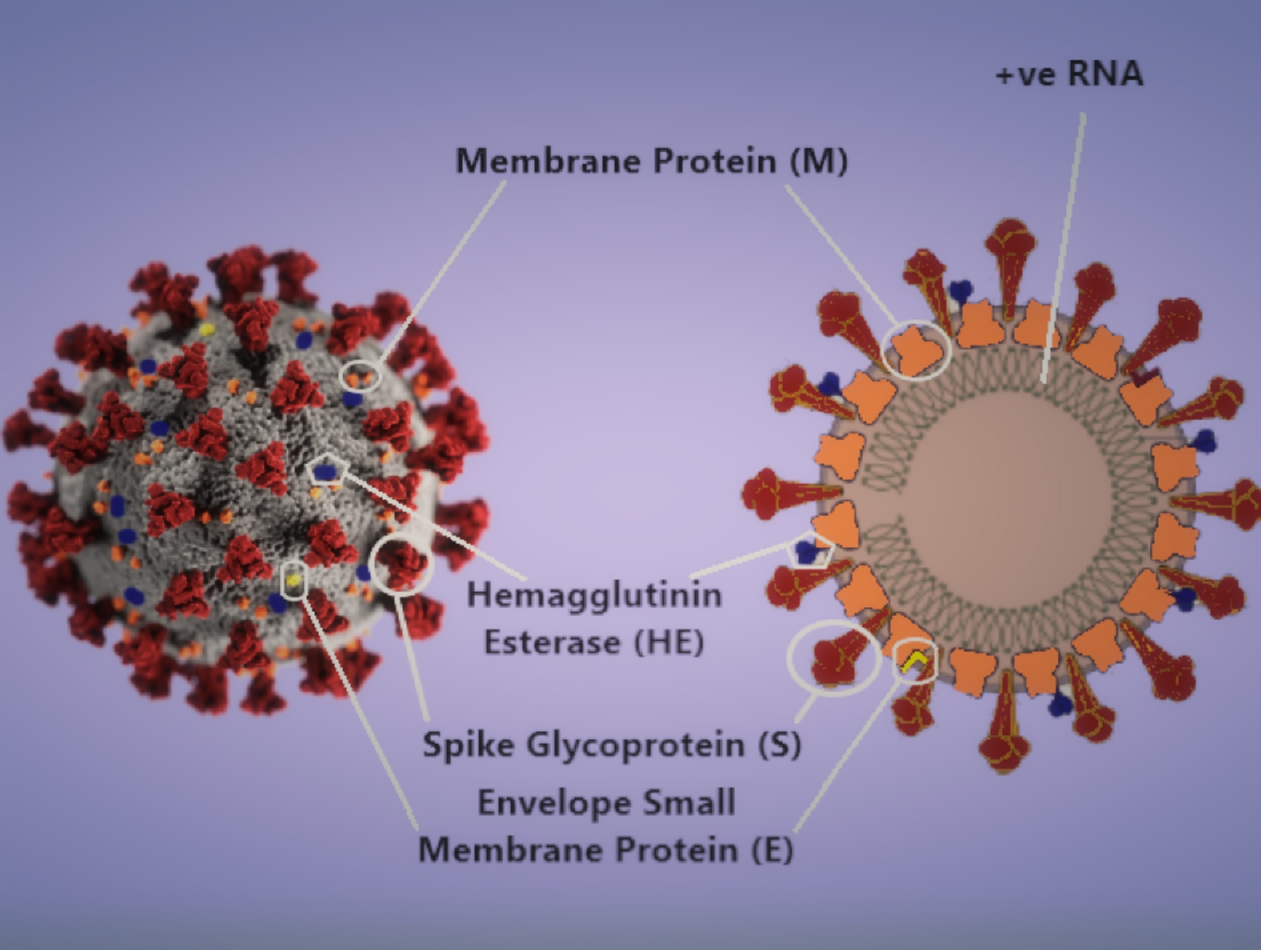
3-D model of the SARS-CoV-2 virion and a schematic diagram of its structural proteins and genome. Image component modified from CDC Public Health Image Library (ID 23312: Alissa Eckert and Dan Higgins)

SARS-CoV2 is from the beta Coronavirus family, it is a positive-sense, single-stranded RNA, enveloped virus that is 50-200 nm in diameter [3]. The genomic RNA is 30 Kb, one vital encoded structural protein is the Spike Glycoprotein (S) that consists of three S1-S2 heterodimers that bind to angiotensin-converting enzyme 2 (ACE2) receptor on type II pneumocyte [3,4]. The other surface protein such as the hemagglutinin-esterase (HE) dimer is shown in Figure 1. The entry of SARS-CoV-2 into the type II pneumocyte is via endocytosis and then multiplies in the cytoplasm. The high protein manufacturing stress induced upon the type II pneumocytes leads to apoptosis. Additionally, the RNA from the SARS-CoV-2 acts as a pathogen-associated molecular pattern (PAMP) and will be recognized by the pattern recognition receptor or toll-like receptors. This leads to a chemokine surge which causes neutrophil migration and activation. This leads to the destruction of the alveolar-capillary walls. At a microscopic level, this leads to a loss in the interface between the intra-alveolar space and the surrounding stroma. Therefore, fluid leaks through and fills into the alveolar sacs.

Origin of SARS-CoV-2

**Figure 2:**
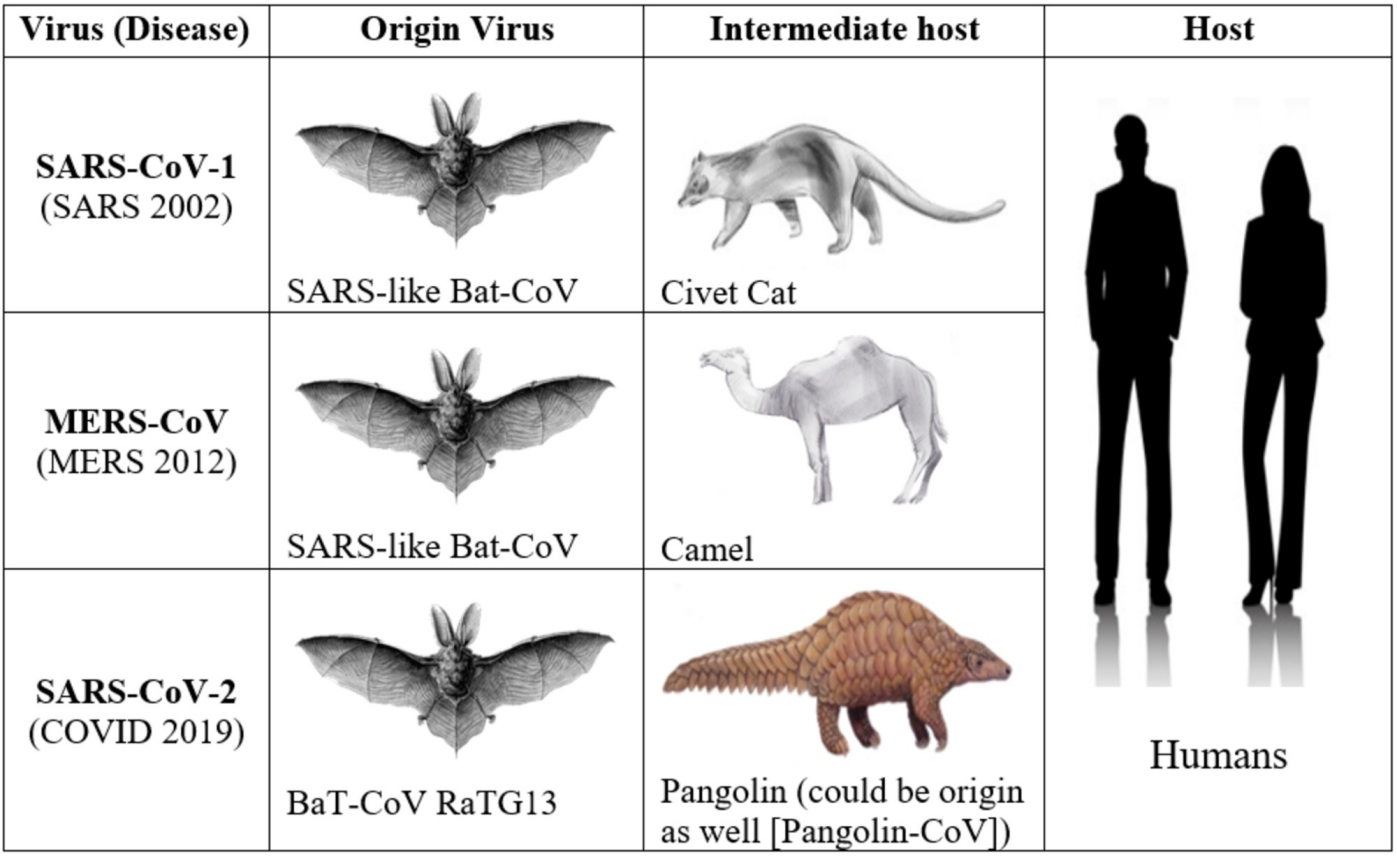
Summary of the natural reservoir, intermediate host and target in major coronaviruses.

The origin of the SARS-CoV-2 genome has been linked to bats akin to the SARS-CoV-1 and MERS-CoV viruses [5]. Interestingly, the SARS-CoV-2 whole-genome aligned with the genomes of viruses (Bat-CoV and Bat-CoV RaTG13) in Rhinolophus affinis species of Yunnan province with 96% similarity [6]. As seen previously in SARS-CoV-1 and MERS-CoV viruses that undertake residence in the intermediate hosts shown in Figure 2, it was suspected that in SARS-CoV-2 pangolins were the natural reservoir. This was based on the analysis of the genome contig alignment of SARS-CoV-2 like CoV (Renamed: Pangolin-CoV) harbored in the lung tissue of two dead Malayan pangolins [7]. This Pangolin-CoV’s whole genome had 91.02% similarity with SARS-CoV-2 and 90.55% similarity with Bat-CoV RaTG13 [8]. Proteomic analysis revealed that the S1 subunit of Spike glycoprotein (S) was more closely related to that of SARS-CoV-2 compared to BaT-CoV RaTG13. Furthermore, five amino acid residues of the S protein of SARS-CoV-2 interacting with the ACE2 receptor are identical in Pangolin-CoV [8]. Contrastingly, only four amino acid residues are identical in the S protein of BaT-CoV RaTG13. Interestingly, both Pangolin-CoV and BaT-CoV RaTG13 have lost the furin recognition motif, vital to the S1/S2 cleavage [8]. This putative furin recognition sequence is still intact within the SARS-CoV-2. A compilation of all these findings portrays that pangolins are the intermediate hosts for SARS-CoV-2.

Evolution of SARS-CoV-2

The phylogenetic analysis by Tang et al. of 103 genomes with SARS-CoV-2 indicated that the two major lineages co-exist. These lineages are designated as L-type (T28,144 is in the codon of Leucine) and S-type (C28,144 is in the codon of Serine) and these are defined by the significant linkage (r2 = 0.954, and LOD = 50.13) of their SNPs at positions 8782 (orf1ab: T8517C, synonymous) and 28144 (ORF8: C251T, S84L) [9]. Furthermore, the S-type (28.7%, n = 29 out of 101) is indicated as the ancestral strain which attained a single-nucleotide polymorphism (SNP) that led to the formation of the L-type (71.9%, n = 72 out of 101). The resultant amino acids from the SNPs in the open reading frame (ORF8) have an undefined functional role in the SARS-CoV-2 life cycle and virulence. Therefore, Tang et al.’s initial labeling of the L-type as the aggressive lineage based on its higher frequency was misleading. This aggressive lineage label has now been removed in their current addendum and the L-type is only defined as having a higher frequency. It must be noted that the 30% and 70% frequencies of the S-type and L-type correspond to patients in Wuhan. Moreover, although the S-type is ancestral lineage it was surprising that the majority of the early cases in Wuhan were of the L-type lineage and globally the S-type is more prominent. One patient (USA_2020/01/21.a, GISAID ID: EPI_ISL_404253) in the USA tested positive for coinfection with S-type and L-type SARS-CoV-2, but it is unclear if this will cause any significant clinical severity due to the coinfection. It is also notable that there is no current evidence indicating that immunity against one of the lineages will provide cross-reactivity against the other lineage. This implies that a patient recovering from one COVID-19 lineage can sustain another separate COVID-19 infection from the other strain. Therefore, future vaccines must target a conserved region in both lineages. Alternatively, a separate vaccine for each lineage must be designed.

Mode of transmission

Modes of transmission traced in an imported case are through droplet transmission, fecal-oral route, conjunctiva and fomites [10, 11]. Additionally, local transmission can be traced back to the patient’s bodily fluids such as respiratory droplets, saliva, feces, and urine [11]. The virion is stabilized at lower temperatures, i.e., 4°C has higher survival than 22°C [12, 13]. As SARS-CoV-2 virions are shed throughout the clinical course, patients with COVID-19 can spread the infection prior to symptom presentation, during the symptomatic course and during the clinical recovery period. Additional considerations must be made regarding the residence time of the SARS-CoV-2 virion on surfaces. The half-life of SARS-CoV-2 in aerosols, copper, cardboard, stainless steel, and plastic are 1.5 h, 1 h, 3.4 h, 5.6 h, and 6.8 h, respectively. The viable residence time of SARS-CoV-1 in aerosols, copper, cardboard, stainless steel, and plastic are 3 h, 4 h, 24 h, 48 h, and 72 h, respectively [14].

Differences between COVID-19, common cold, and flu

Common cold is caused by a myriad of viruses. Majority of which are Rhinoviruses, and benign forms of coronaviruses. Common cold and COVID-19 both have a gradual course to symptom presentation in comparison to the flu which is caused by the various strains of Influenza (Orthomyxovirus family). Pyrexia is rare in the common cold but is the most notable symptom in both COVID-19 and flu [15]. Presentation of cough and fatigue is rare in the common cold. Coryzal symptoms such as rhinorrhea and nasal congestion are predominant in the common cold and are rare in flu and COVID-19.

COVID-19 presents similar to Influenza flu as both these are diseases of the respiratory system. In both diseases, the clinical presentation can vary from asymptomatic to severe pneumonia. Furthermore, both COVID-19 and Influenza are transmitted by contact, droplets, and fomites. Therefore, similar hand hygiene techniques and respiratory etiquettes will be beneficial in preventing the spread. Another factor that influences the rate of spread of any infection is the Basic Reproduction Number (Rₒ). The influenza virus has an Rₒ of ~1.3 whereas the SARS-CoV-2 virus has an Rₒ of ~2.3. Therefore, each COVID-19 patient can spread 1.8-fold more new contacts compared to influenza patients.

In comparison to SARS (caused by SARS-CoV-1 virus), some patients with COVID-19 (caused by SARS-CoV-2 virus) can be infectious during their incubation period even in their asymptomatic stage [14]. The time elapsed from the advent of exposure to a pathogen to clinical manifestation of the disease is termed as the incubation period. Table 1 shows the variance in the incubation periods of each coronavirus and common orthomyxovirus. The larger incubation for the manifestation of COVID along with the ability to transmit infection during this period explains how rapid the potential spread of SARS-CoV-2 can be.

**Table 1:** Summary of incubation times of various coronaviruses and orthomyxovirus.

Virus Family	Virus (Disease)	Incubation Period	References
Coronavirus	SARS-CoV-2 (COVID-19)	2-14 days	[16]
	SARS-CoV-1 (SARS)	2-7 days	[17]
	MERS-CoV (MERS)	5 days	[18]
Orthomyxovirus	H1N1 Influenza A (swine flu)	1-4 days	[19]
	Influenza A (Seasonal flu)	2 days	[15]

## Review

Clinical presentation of COVID-19

The infection caused by the virus SARS-CoV-2 is termed as Coronavirus Disease 2019 (COVID-19). The symptomatology of COVID-19 was extensively discussed in WHO-China joint report on COVID-19 (n = 55,924) [16]. Patients with COVID-19 present with pyrexia in 85% of cases during their illness course, but only 45% are febrile on early presentation [20]. Moreover, cough is seen in 67.7% of patients and sputum is produced in 33.4%. Respiratory symptoms such as dyspnea, sore throat, and nasal congestion present in 18.6%, 13.9%, and 4.8% of cases, respectively [20]. Constitutional symptoms such as muscle or bone aches, chills, and headache are seen in 14.8%, 11.4% and 13.6% of the cases, respectively [20]. Gastrointestinal (GI) symptoms such as nausea or vomiting and diarrhea are seen in 5% and 3.7% of the cases, respectively. These clinical manifestations of COVID-19 were consistent in other similar studies on COVID-19 patients in China (n = 41, n = 81, n = 99, n = 138) [21-24].

More severe insult on the lung tissue can result in acute respiratory distress syndrome (ARDS) which can further precipitate septic shock. These two complications are the major contributors to intensive care unit (ICU) care and mortality from COVID-19 in patients older than 60 years, with smoking history, and comorbid medical conditions. Smoking and older age group patients tend to have a higher density of ACE2 receptors. A list of chronic medical conditions affecting the clinical course of COVID-19 is summarized in Table 2. Our overall analysis (N = 1458) showed that the leading comorbid conditions include hypertension, cardiovascular and cerebrovascular disease, and diabetes.

**Table 2:** Summary of co-morbidity history from COVID-19 patients in China. CAD: Coronary artery disease; CVA: Cerebrovascular accident; GI: Gastrointestinal; CNS: Central nervous system; COPD: Chronic obstructive pulmonary disease; CRF: Chronic renal failure.

	Guan et al. (N = 1099) [20]	Wang et al. (N = 138) [21]	Chen et al. (N = 99) [22]	Shi et al. (N = 81) [23]	Huang et al. (N = 41) [24]	Analysis N = 1458
Comorbidity	23.7% (n = 261)	46.4% (n = 64)	51% (n = 50)	26% (n = 21)	32% (n = 13)	28% (n = 409)
CAD and CVA	3.9% (n = 42)	19.6% (n = 27)	40% (n = 40)	17% (n = 14)	15% (n = 6)	31.5% (n = 129)
Hypertension	15% (n = 165)	31.2% (n = 43)	-	15% (n = 12)	15% (n = 6)	55.3% (n = 226)
GI disease	-	-	11% (n = 11)	-	-	2.7% (n = 11)
Diabetes	7.4% (n = 81)	10.1% (n = 14)	12% (n = 12)	12% (n = 10)	20% (n = 8)	30.6% (n = 125)
Malignancy	0.9% (n = 10)	7.2% (n = 10)	1% (n = 1)	5% (n = 4)	2% (n = 1)	6.4% (n = 26)
CNS diseases	-	-	1% (n = 1)	-	-	0.2% (n = 1)
COPD	1.1% (n = 12)	2.9% (n = 4)	1% (n = 1)	11% (n = 9)	2% (n = 1)	6.6% (n = 27)
CRF	0.7% (n = 8)	2.9% (n = 4)	-	4% (n = 3)	-	3.7% (n = 15)
Immunodeficiency	0.2% (n = 2)	1.4% (n = 2)	-	-	-	1% (n = 4)
Hepatitis/ Liver Cirrhosis	21% (n = 23)	2.9% (n = 4)	-	9% (n = 7)	2% (n = 1)	8.6% (n = 35)

In the largest study (n = 1099), the median age of the patients with COVID-19 is 47 years [20]. The presentation of COVID-19 is predominantly mild and asymptomatic in the age group <14. There is no explanation for this phenomenon yet. Perhaps the ACE2 receptor is not highly expressed in this age group. A preliminary unpublished single-cell transcriptomics study suggested that Asian cell donors have higher ACE2 receptor density than Caucasian and African American donors. This variance in distribution could explain the higher susceptibility of the Asian population to the SARS-nCoV-2 virus.

Clinical disease course of COVID-19

The official incubation period for SARS-CoV-2 is 2-14 days and therefore 14 days is the chosen cut-off for self-quarantine. Guan et al. (n = 1324) estimated that the mean incubation period is three days [95% CI: 2 to 7 days] and that in 95% of all COVID-19 patients developed illness onset within 10 days [20]. Outlier cases have been found wherein the incubation period has been 19, 24, and 27 days.

Wang et al. (n = 138) showed that the median time from preliminary symptoms to dyspnea, hospital admission and ARDS was 5, 7, and 8 days, respectively. The median hospital stay was 10 days in patients that were discharged [21].

**Figure 3:**
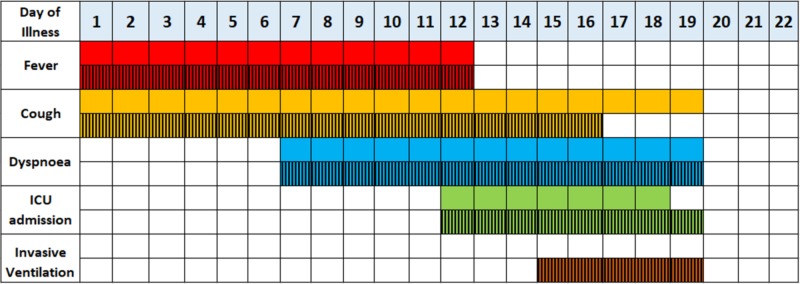
Summary of the clinical course of COVID-19 patients. The solid colors and cross-hatched patterns indicate the survivors (n = 137) and non-survivors (n = 54), respectively.

Figure 3 shows a comprehensive summary of the clinical course of COVID-19 patients (n = 191) and this is stratified based on survivors and non-survivors [25]. Zhou et al. showed that the survivors group developed ARDS and sepsis on days 9 and 10, respectively. Conversely, the non-survivors group developed ARDS and sepsis later, on days 10 and 12, respectively. Furthermore, the non-survivors group developed more complications such as acute kidney injury (AKI) and secondary infection by days 15 and 17, respectively. Lastly, the survivor groups were discharged from the hospital by day 22 and the non-survivor groups died by day 19.

Epidemiology

As of March 21, there have been 304,900 cases of which 94,793 have recovered and 13,001 have died [26]. Therefore, the worldwide case fatality rate (CFR) within this time period is 12%. The current estimate of COVID-19 Rₒ is 2-2.5 [16, 27]. This can be interpreted as every case of COVID-19 can spread to two to three new people. Table 3 shows a comparison between the epidemiological parameters of the major infectious outbreaks from 2000-2020 [19, 28, 29].

**Table 3:** Summary of cases, mortality rate, and basic reproductive number of major outbreaks from 2000-2020. *Accurate as of March 22, 2020

Virus (Disease)	Cases	Mortality Rate	Rₒ
SARS-CoV-2 (COVID-19)	304,900*	3.4% estimated from WHO on March 3, 2020	2-2.5
SARS-CoV-1 (SARS 2020)	8,098	9.6%	2-5
MERS-CoV (MERS 2012)	2,494	34%	0.3-0.8
H1N1 Influenza A (Swine flu 2009)	60.8 million	0.02%	1.4-1.6

**Figure 4:**
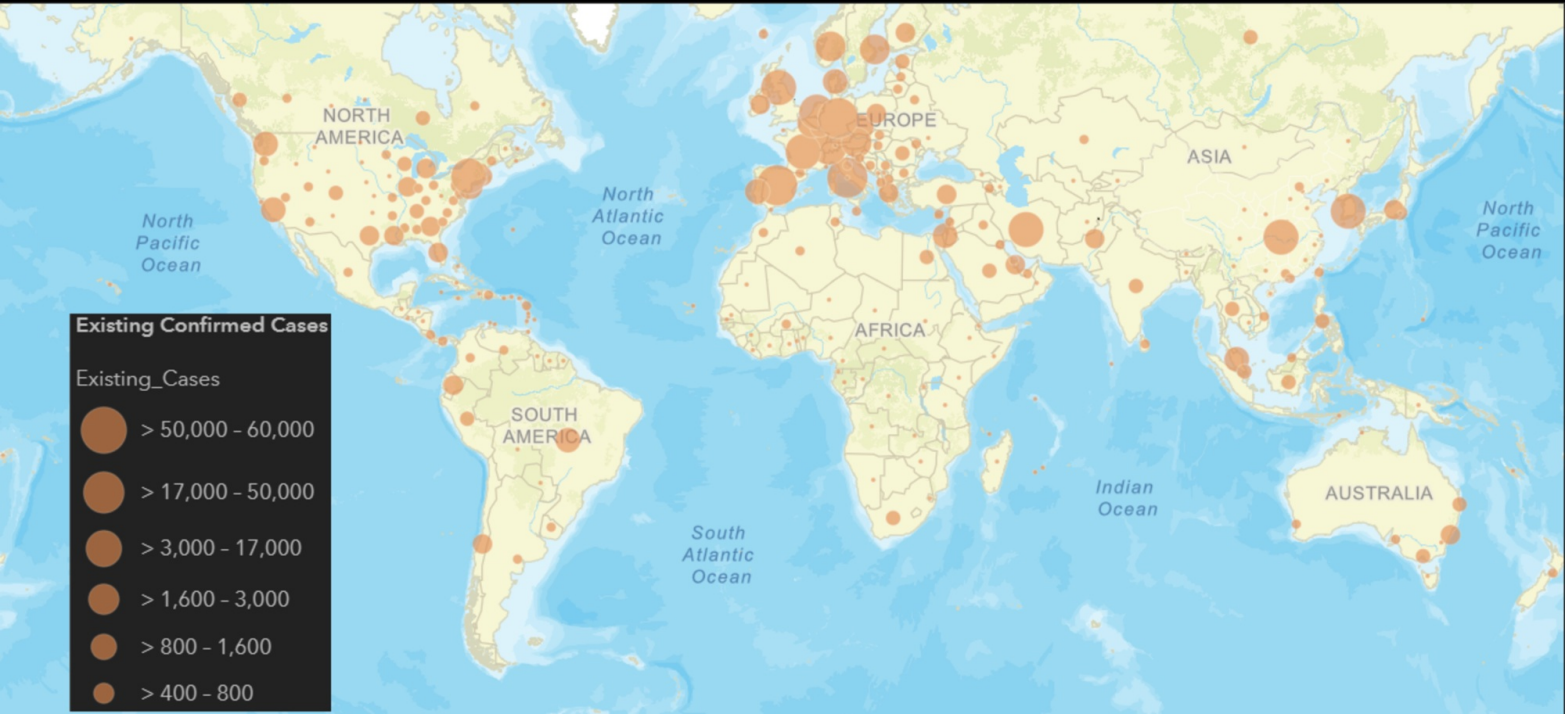
Illustration of the geographical spread of active COVID-19. This data is accurate as of 22nd March 2020. The image is modified from the source [30].

As shown in Figure 4, a total of 188 countries and territories along with the Diamond Princess cruise ship (currently harbored in Japan) have been affected [26, 30]. The major cluster of active cases has been in the USA, Italy, Spain, Germany, and France.

Countries with the highest mortality rates are Italy, Spain, Iran, France, and the USA [30]. Mortality rate increases in the 60 years and above cohort to 8.8% in comparison to 0.46% for patients under 60 years old [16]. The mortality rate has a predilection for the male gender (M:F = 1.7:1). Furthermore, the mortality rate also increases in patients with additional comorbidities. The cardiovascular, diabetes and hypertension leading the cohort with mortality rates of 13.2%, 9.2%, and 8.4%, respectively [31].

Screening tool

There are two prominent screening tools, one developed by the CDC and the other by the WHO. Both criteria charts have evolved over the progression of the outbreak. Table 4 is the most updated COVID-19 screening tool developed by the CDC [32].

**Table 4:** Summary of CDC COVID-19 screening criteria.

Clinical Features		Epidemiologic Risk (within 14 days of symptom onset)
Pyrexia OR Respiratory symptoms (cough, dyspnea, sore throat, and nasal congestion)	AND	Close contact with RT-PCR confirmed COVID-19 patient
Pyrexia AND Respiratory symptoms (cough, dyspnea, sore throat, and nasal congestion) requiring hospitalization	AND	History of travel to CDC flagged areas
Pyrexia AND Severe Respiratory illness (pneumonia, ARDS) requiring hospitalization AND without any alternative diagnosis	AND	No discernment of exposure history

Diagnostic tools

On 12th January 2020, the China CDC shared the genetic sequence of the SARS-CoV-2. This enabled countries to develop primers against the SARS-CoV-2 genome and utilize reverse transcriptase polymerase chain reaction (RT-PCR) assays to make a diagnosis of COVID-19. Therefore, RT-PCR has become the gold standard for the diagnosis of COVID-19, but it is only 66-80% sensitive [33]. Essentially, this means that 20-34% of patients with COVID-19 out of 100 would test negative despite being infected. This variance in the sensitivity can be attributed to the patients being tested early in the disease course wherein the viral load is beneath detection level or due to lack of automation in sample preparation for RT-PCR. Furthermore, a single negative RT-PCR does not rule out COVID-19, hence a repeat RT-PCR must be performed. The concern rises regarding the timeframe of the repeat RT-PCR, the ideal window lies between 24 to 72 hours of the negative test.

The largest study (n = 1014) showed that CT-chest had a 95% sensitivity in making an early diagnosis of COVID-19 through the identification of ground-glass opacities [33]. This can be interpreted as five out of 100 tested will be falsely ruled out. This finding was also consistent in a case series of 51 COVID-19 patients wherein the sensitivity of CT was 98% and RT-PCR was 71% (p < 0.001) for patients investigated with imaging and assay within three days of admission [33]. The utilization of the CT scan would prevent an infected patient from being discharged back to the community. The cons of increased CT usage are the huge economic burden on the healthcare resources and the potential to contaminate the CT scanners. In the management algorithm, CT radiology can be used to stratify patients into those that require further investigation and isolation. Utilization of radiology can be directive for management plans in hospitals with ease of accessibility to CT radiology and lack of access to laboratory services or in countries that utilize a centralized laboratory service with large turnaround time.

Contrastingly, chest X-rays have poor sensitivity, but if there are ground-glass opacities then the patient must be isolated. The pathophysiology of this radiological finding is that the SARS-CoV-2 virus induces an inflammatory response in the alveolar sacs leading to the accumulation of exudative fluid. In the severe progression of the disease, this moves to the lobes and the radiological finding is a solid white consolidation.

Laboratory examination

Standard blood investigations revealed that most patients with COVID-19 have normal or decreased leucocytes, and lymphocytopenia. Furthermore, there is a systemic elevation of the pyrogenic cytokines such as IL-6, IL-10, and TNF-α [20-24]. In critical COVID-19 patients, neutrophilia, elevated D-dimer, increase in plasma blood urea nitrogen (BUN) and creatinine are also documented [20-24, 33]. Patients admitted to the ICU will also have elevated plasma levels of the interleukins (IL-2, IL-7, and IL-10), and other chemokines such as Granulocyte Colony Stimulating Factor, 10 kD Interferon-gamma-induced-protein-10, Monocyte Chemoattractant Protein-1, and Macrophage Inflammatory Protein 1-α [24].

Management

The management of viral pneumonia is predominantly supportive amidst the absence of validated antiviral drugs. The most primary symptoms managed in COVID-19 are fever and non-productive cough, therefore the first-line antipyretic agent is Paracetamol and antitussive of choice is guaifenesin [21]. Supplementary oxygen at 5 L/min must be administered for patients that require management of severe respiratory distress and the oxygen saturation (SaO2) target must be ≥92-95% in pregnant patients and ≥90% in all other patients [34]. Complications such as septic shock and AKI should be managed with sepsis protocol and renal replacement therapy (RRT) respectively. Renal function tests and fluid balance measurements will enable the identification of patients indicated for RRT [21]. Some patients may develop superimposed bacterial or fungal infection in the middle to later course of COVID-19, as such appropriate empiric antimicrobial coverage must be provided. The latest version (6th edition) of the Guidelines for the Prevention, Diagnosis, and Treatment of COVID-19 by the National Health Commission (NHC) of China has recommended a combination regimen of protease inhibitors (lopinavir and ritonavir) with INF-α. The rationale for this combination treatment is based on experience with this regimen in reducing the mortality rates in SARS. The WHO recommends usage of extracorporeal membrane oxygenation (ECMO) in patients that sustain hypoxia refractory to supplementary oxygen [31]. Alternatively, convalescent plasma and IgG are used as rescue therapy in critical cases but there is no robust evidence for this practice.

In the majority of the cases, public health measures are vital for the management of the spread of COVID-19. If public health measures for containment are not adequate, then there will be a patient burden that supersedes the capacity of available ICU beds and mechanical ventilation, as seen in the crisis taking place in Italy. Therefore, the entire objective of the COVID-19 management rests on the premise of social distancing to suppress the rapid emergence influx of new cases in a short time frame. This epidemiological concept is referred to as the “flattening of the curve”. The mainstay of public health must be to identify the infective cases, isolate these cases, attain contact tracing and isolate contacts that present with symptoms.

Discharge criteria and quarantine discontinuation

Four major discharge criteria exist, and these are from Italy (Ministero della salute, Consiglio Superiore di Sanità), China (China CDC), USA (CDC), and Singapore (National Centre for Infectious Diseases). These models differ only in their cutoffs.

The China CDC discharge criteria state that all four conditions must be met to satisfy a discharge from the hospital [35].

1. A patient must remain apyrexic for at least three consecutive days.

2. All respiratory symptoms (cough, dyspnea, sore throat, and nasal congestion) must be resolved.

3. Chest CT must demonstrate marked resolution of the exudative lesion.

4. Two serial RT-PCRs must be negative for SARS-CoV-2 RNA from the nasopharyngeal collection, these assays must be spaced by 24 hours.

Quarantine discontinuation criteria

Two separate quarantine discontinuation criteria for COVID patients in self-quarantined at home have been developed by Italy (Ministero della salute, Consiglio Superiore di Sanità) and USA (CDC).

The CDC quarantine discontinuation criteria state that both conditions must be met to satisfy the criteria [35].

1. At least two serial RT-PCRs must be negative for SARS-CoV-2 RNA. These swabs must be nasopharyngeal collections, these assays must be spaced by 24 hours.

2. The patient must remain apyrexic for at least 72 hrs without antipyretic medication use, and resolution of respiratory signs and symptoms. A minimum of seven days have passed since the preliminary symptom appeared.

Prevention strategies

Self-Protection

Hand washing for at least 20 seconds after visiting public spaces. Soap or hand sanitizer with at least 60% of ethanol is recommended [36]. It is also recommended to avoid touching the denoted facial T-zone (eyes, nose, mouth) as this is the access point for virions into the upper respiratory tract [36]. Avoiding contact with people who are already presenting with symptoms, as well as avoiding gathering or crowded places. Travel to outbreak areas must be prohibited. A healthy individual must maintain at least six feet distance from individuals presenting with symptoms [36]. The sterilization of frequently handled surfaces is beneficial.

All healthcare workers managing COVID-19 patients require full personal protective equipment (PPE) containing surgical masks, double gloves, full-sleeved procedural gowns, and eye shield [36]. The N95 masks which prevent 95% of the droplets from entering the mask must be exclusively dawned prior to performing procedures associated with a higher risk for aerosol exposure such as tracheostomy, tracheal intubation, bronchoscopy, cardiopulmonary resuscitation (CPR), and noninvasive ventilation (NIV) [36]. These procedures have the potential to aerosolize the virus.

Containment of community transmissions is achieved by the closure of educational institutions, businesses, airspace, and sports events. High-risk individuals such as those older than 65 or having chronic comorbidities without any symptoms are also required to self-quarantine to decrease the likelihood of COVID-19 contraction [36].

Herd Protection

On the development of any symptoms, the potential patient should remain quarantined in self-isolation away in a separate room with a separate bathroom for at least 14 days. This self-isolation must be extended to pets as well, as there is a recorded case of a human-to-dog transmission [31]. If there are any further concerns about COVID-19, then immediate contact with the public health hotline or general practice clinic via telemedicine must be established to attain a potential diagnosis. Face masks (N95) are needed for COVID-19 patients to prevent droplet spread [31].

Role of vitamins

Vitamin C

Vitamin C (L-ascorbic acid) has a pleiotropic physiological role, but there is evidence supporting the protective effect of high dose intravenous vitamin C (HDIVC) during sepsis-induced ARDS. Vitamin C reinforces the maintenance of the alveolar epithelial barrier and transcriptionally upregulates the protein channels (CFTR, aquaporin-5, ENaC, and Na+/K+ ATPase) regulating the alveolar fluid clearance [37]. HDIVC has been implicated in reducing plasma cell-free DNA formed from the neutrophil extracellular trap (NET) which is the facilitator of systemic inflammation in sepsis-induced multi-organ failure [38,39]. Interestingly, elevated levels of syndecan-1 in the plasma correlate with increased mortality in severe sepsis and ARDS, and this endothelial glycocalyx can be reduced significantly by HDIVC [39]. As of 14 February 2020, there is a randomized controlled trial (RCT) undertaken at the Zhongnan Hospital (NCT04264533) that aims to evaluate the clinical efficacy and safety of vitamin C in viral pneumonia from SARS-CoV-2. They hypothesize that vitamin C infusion can improve the prognosis of severe acute respiratory tract infections. The treatment arm includes a 12 g vitamin C infusion (q12h) for seven days and the primary outcome measures the ventilation-free days. The estimated completion time is September 2020.

Vitamin D

Vitamin D is known to mitigate the scope of acquired immunity and regenerate endothelial lining. This may be beneficial in minimizing the alveolar damage caused in ARDS. Level I evidence (N = 11,321) showed that there is a 12% overall protective effect of vitamin D supplementation against bacterial and viral acute respiratory tract infection (adjusted OD = 0.88, p < 0.001) [40]. These protective effects increased to 19% in those individuals on the daily or weekly regimen of vitamin D compared to those dosing on a monthly bolus of vitamin D (adjusted OD = 0.81, p < 0.001). Furthermore, there is a 70% protective effect when vitamin D deficiency is corrected with supplementation (adjusted OD = 0.30, p = 0.006) [40]. This result is pertinent to the majority of individuals residing in the northern latitudes that experience vitamin D deficiency (serum 25-hydroxyVitamin D <25 nmol/L) due to extended periods of lack of sunlight.

Vaccination

Unfortunately, there is no approved vaccine against COVID-19 as of March 2020. One of the leading candidates is the mRNA-1273 vaccine manufactured by ModernaTx Inc (Cambridge, MA, USA). The mRNA-1273 is encapsulated within a lipid nanoparticle and encodes for a full-length, prefusion stabilized spike glycoprotein (S) of the SARS-CoV-2 virus. This vaccine is currently in Phase I, Open-Label, Dose-Ranging, clinical trial (NCT04283461) to evaluate the safety profile, reactogenicity, and immunogenicity in healthy subjects. The estimated completion time is June 2021.

Novel therapeutics

Currently, multiple avenues for therapies are being explored. Summarized below are some of the more prominent candidates.

Remdesivir

Remdesivir (GS-5734) is a nucleoside inhibitor that is the strongest candidate from COVID-19 treatment. Remdesivir is a monophosphoramide prodrug that causes premature termination of viral RNA replication and was originally developed against Ebola, MERS-CoV, and SARS-CoV. Furthermore, an in vitro study on human cell line (human liver cancer Huh-7 cells) showed potent interference of remdesivir with the NSP12 polymerase of SARS-CoV-2 despite intact ExoN proofreading activity [41]. There are eight clinical trials currently underway in China (NCT04252664, NCT04257656), France (NCT04314817, NCT04315948) and the USA (NCT04315948, NCT04292730, NCT04280705, NCT04302766). The suggested dosing is for a 10-day course, IV 200 mg for the first day and then IV 100 mg for nine following days.

Lopinavir and Ritonavir

The protease inhibitors lopinavir and ritonavir combination is usually a part of the HAART regimen to treat HIV. The lopinavir and ritonavir combination has also been shown to be effective against SARS in vitro [42]. The current Chinese guidelines for COVID-19 treatment include a PO 50 mg-200 mg dose BID for a duration of 10 days [43]. The lopinavir and ritonavir are used as a regimen single-agent or combination with either ribavirin or interferon-α. Currently, an RCT (Chinese Clinical Trial Register number: ChiCTR 2000029308) with 199 patients in Wuhan aims to evaluate the safety and efficacy of lopinavir and ritonavir regimen in severe COVID-19 patients. This was a two-arm study comparing lopinavir-ritonavir (n = 99) to standard care (n = 100). There was a statistically significant difference in the time to clinical improvement between the two groups on day 14 but this result was not statistically significant on day 28. The mortality at 28 days reduced by 5.8% and the length of stay in the ICU reduced by five days with the lopinavir-ritonavir treatment [44]. There are 12 clinical trials currently underway in South Korea (NCT04307693), Thailand (NCT04303299), and China (NCT04286503, NCT04255017, NCT04261907, NCT04261907, NCT04295551, NCT04315948, NCT04275388, NCT04251871, NCT04276688, NCT04291729, NCT04306497).

Umifenovir

Umifenovir is a non-nucleoside broad-spectrum antiviral licensed for influenza treatment and prophylaxis in Russia and China. It has not received FDA approval yet. Umifenovir is a membrane fusion inhibitor. Current regimens of Umifenovir used in China include a PO dose of 200 mg TDS for a duration of 10 days [43]. One large Chinese RCT (GDCT0379047) aims to evaluate the safety and efficacy of combination treatment of Umifenovir with immunostimulatory recombinant cytokine gene-derived protein. Another Chinese RCT (NCT04246242) aims to evaluate the safety and efficacy of single-agent treatment of Umifenovir.

Chloroquine

Chloroquine is an anti-malarial medication. In viruses, chloroquine can inhibit pH-dependent stages of replication. Furthermore, chloroquine’s immunomodulation is dependent on the suppression of cytokines (IL-6 and TNF-α) production and dissemination. Moreover, experiments with monkey cell line (Vero E6) showed that chloroquine interferes with the receptor glycosylation and thereby affects the entry mechanism of SARS-CoV-2. This treatment was especially successful in the in vitro experiments with SARS-CoV-2 infection of a human cell line (human liver cancer Huh-7 cells) [41]. Interestingly, pharmacological modeling utilizing dosing from another in vitro study showed that hydroxychloroquine has a higher potency than chloroquine at inhibiting the SARS-CoV-2 infection. The suggested dosing from this study was PO hydroxychloroquine 400 mg BID for the first day and then 200 mg BID for the following four days [45].

Secondary COVID-19 rates can be minimized with pre-exposure prophylaxis and post-exposure prophylaxis in an individual with document exposure to SARS-CoV-2. Therefore, hydroxychloroquine has been hypothesized to be an adequate chemoprophylaxis candidate to reduce secondary COVID-19. There are six clinical trials currently underway in Mexico (NCT04315896), South Korea (NCT04307693), China (NCT04261517, NCT04307693), Spain (NCT04304053), Norway (NCT04316377), and USA (NCT04308668). The first Chinese study (NCT04261517) has shown positive outcomes in its preliminary data. Although the sample size is small (n = 30), this is still promising. The current Chinese guidelines recommend dosing of chloroquine as PO 300 mg or 500 mg (Chloroquine phosphate) BID for a duration of 10 days [43].

ACE Inhibitor (ACEi) and Angiotensin Receptor-1 Blocker (ARBs)

SARS-CoV-2 enters the type II pneumocytes via the ACE2 receptor, and this is also a functional receptor. The functional role of the ACE2 receptor has a reciprocal physiological action to ACE1, it converts the angiotensin II back into angiotensin I. Therefore, patients taking ARBs will have an increased plasma level of angiotensin II. Contrastingly, patients taking ACEi will have low levels of angiotensin II. There is an upregulation of ACE2 receptors in the kidney and heart in response to ACEi or ARB dosing in rats and humans [46-48]. There is no data available on its effect in the alveolar tissue. If there is a similar upregulation of ACE2 receptors then there will be heightened infectivity of SARS-CoV-2 along with subsequent clinical illness severity. Discontinuation of ACEi or ARBs is not recommended yet as hypertension is an acute risk of discontinuation and can exacerbate the clinical course and increase mortality of COVID-19 if infected by SARS-CoV-2. This view of not discontinuing ACEi and ARBs has been supported by the council on hypertension from the European Society of Cardiology.

Antipyretics

Ibuprofen has shown to upregulate ACE2 receptors [49]. There is no current evidence indicating that ibuprofen worsens the clinical course of COVID-1. The current standpoint of the WHO is to continue the use of ibuprofen as antipyretic agent. The first-line antipyretic remains to be acetaminophen.

Systemic Corticosteroids

The use of systemic corticosteroids such as glucocorticoid in the management of ARDS secondary to viral pneumonia is controversial. The rationale behind this approach is that the corticosteroids prolong the viral shedding time and maintain a systemic anti-inflammatory state that will minimize the precipitation of ARDS, dyspnea, and severe pneumonia. The WHO states that the use of corticosteroid is not recommended outside of clinical trials (NCT04273321) or otherwise indicated [31]. Moreover, the MERS patients that received systemic corticosteroids had a higher likelihood of receiving invasive mechanical ventilation and a raised mortality rate at day 90 [50].

## Conclusions

This COVID-19 pandemic is a reminder of the volatility in the ongoing planning to manage the primary and secondary infection of SARS-CoV-2. This planning can be improved by accurate modeling of current data and by eliminating the misinformation in our era of data surplus. Additional variables that can strengthen countermeasure to this pandemic are rapidly updating surveillance data, availability of robust accredited information, and a multidisciplinary approach that bridges the gap in knowledge between basic sciences and clinical sciences.

This literature review comprehensively summarizes the most relevant study relating to the individual parameters that influence the clinical course and management of COVID-19. Due to the lack of available and validated therapeutics, most of the countermeasures rely on the usage of public health containment and quarantine approaches. Primary learning points from this COVID-19 pandemic are to upheld transparency to prevent delays in threat identification. Secondly, delays in travel restriction and self-quarantine measures led to a logarithmic expansion of cases. Lastly, there is a need to increase investments towards research and development in COVID-19.
